# Membrane proteomics and transcriptomic profiling analysis of hepatic stellate cells co-incubated with *Schistosoma japonicum* eggs

**DOI:** 10.3389/fcimb.2025.1674880

**Published:** 2025-09-16

**Authors:** Bowen Dong, Haoran Zhong, Danlin Zhu, Hao Li, Ke Lu, Zhiqiang Fu, Jinming Liu, Yamei Jin

**Affiliations:** National Reference Laboratory for Animal Schistosomiasis, Key Laboratory of Animal Parasitology of Ministry of Agriculture and Rural Affairs, Shanghai Veterinary Research Institute, Chinese Academy of Agricultural Sciences, Shanghai, China

**Keywords:** *Schistosoma japonicum* egg, hepatic stellate cells, surface proteins, mass spectrometry, RNA-sequencing

## Abstract

Schistosomiasis has been recognized as the second most prevalent parasitic disease worldwide, following malaria. Schistosome eggs can persist for extended periods within host hepatic tissues, leading to hepatic fibrosis primarily through the activation of hepatic stellate cells (HSCs). However, the mechanisms by which egg-secreted products modulate the activation of HSCs remain incompletely elucidated. In this study, purified *Schistosoma japonicum* (*S. japonicum*) eggs (Egg group) or corresponding unused medium (Control group) were placed in the upper chamber of a Transwell system, with HSCs cultured in the lower chamber. Following co-culture, HSCs surface proteins were eluted and subsequently analyzed by mass spectrometry. Protein identities were determined by matching spectral data against both human and schistosome protein databases. A total of 88 schistosome proteins, including both *S. japonicum*-specific and non-specific proteins, were identified in the Egg group. Bioinformatic analyses suggested that HSCs were exposed to egg-derived secretory proteins, indicating potential molecular interactions between schistosome eggs and host cells. Furthermore, RNA-sequencing was performed on HSCs following co-culture, resulting in the identification of 634 differentially expressed genes (DEGs), of which 454 were upregulated and 180 were downregulated. Functional enrichment analyses revealed significant involvement of these DEGs in fibrosis- and inflammation-related pathways. Collectively, this study provides novel evidence that *S. japonicum* eggs may remotely modulate the transcriptional profiles of HSCs via secreted bioactive molecules, thus offering a theoretical foundation for identifying potential therapeutic targets in hepatic fibrosis.

## Introduction

Schistosomiasis, primarily caused by the parasitic trematode worms *Schistosoma mansoni* (*S. mansoni*), *Schistosoma japonicum* (*S. japonicum*), and *Schistosoma haematobium* (*S. haematobium*) is endemic in 78 tropical and subtropical countries or regions, affecting over 250 million individuals worldwide according to the World Health Organization (Buonfrate et al., 2025). Infections with *S. japonicum* and *S. mansoni* can lead to severe liver fibrosis. Female worms of *S. japonicum* and *S. mansoni* exhibit a remarkable egg-laying capacity, releasing hundreds of eggs daily. These eggs accumulate primarily in the hepatic sinusoids and branches of the portal vein, becoming the primary pathogenic factor (Liu et al., 2022). Some eggs penetrate the vascular endothelium and migrate into the liver parenchyma (Costain et al., 2018), where the excretory-secretory products (ESPs) secreted by the mature eggs leads to severe hepatic inflammation and aggravates a pathological response characterized by granulomas surrounding the eggs, ultimately progressing to liver fibrosis and cirrhosis (Cando et al., 2022; Zhang et al., 2019).

While the eggs are considered the main causative factor, some *in vitro* studies have found that certain substances derived from the eggs can reduce HSC activation. For example, Anthony et al. (Anthony et al., 2010) demonstrated that through a transwell co-culture system that the ESPs from *S. mansoni* eggs can revert the activated HSCs to a quiescent state. Specifically, the release of ESPs into the extracellular environment resulted in significant downregulation of key fibrotic markers, including α-smooth muscle actin (α-SMA) and type I collagen (Col1α1), while upregulating the expression of peroxisome proliferator-activated receptor-γ (PPARγ), a marker associated with the quiescent state of HSCs (Anthony et al., 2010). Similarly, *S. japonicum* eggs co-cultured with HSCs, either in direct contact or via a transwell system, were shown to significantly inhibit the fibrogenic phenotype of activated HSCs, as evidenced by the reduction in fibrosis-associated markers (Anthony et al., 2013). As research advances, extracellular vesicles (EVs), which possess a bilayer membrane structure and contain nucleic acids, proteins, carbohydrates, and other bioactive molecules (van Niel et al., 2018), have gained attention as a crucial component of ESPs (Murphy et al., 2020). Recent studies have demonstrated that EVs derived from schistosome eggs, as a key component of ESPs, are also involved in modulating host hepatic fibrosis. Notably, the sja-miR-71a from *S.japonicum* egg-derived EVs has been reported to alleviate liver fibrosis (Wang et al., 2020). Despite these findings, the macroscopic regulatory effects of schistosome eggs on HSCs remain incompletely understood.

In this study, a transwell model was employed to co-culture *S. japonicum* eggs with HSCs. Surface proteins of the co-cultured HSCs were extracted and analyzed using mass spectrometry (MS) to identify potential schistosome proteins present on HSCs surface. Subsequently, transcriptomic sequencing was performed to profile the RNA expression of HSCs under the co-culture conditions with the eggs. This approach aims to provide a comprehensive understanding of the impact of egg-derived ESPs on HSCs, thereby providing data support for the identification of potential targets for alleviating liver fibrosis.

## Materials and methods

### Ethics statement

Six-week-old male New Zealand white rabbits, purchased from the Shanghai Laboratory Animal, Co., Ltd. (Shanghai, China), were used for eggs collection. The *S. japonicum* Anhui strain was provided by the Shanghai Veterinary Research Institute, Chinese Academy of Agricultural Sciences (Shanghai, China). The numbers of viable cercariae were determined prior to infection using a light microscope. The protocols of all animal experiments were approved by the Animal Care and Use Committee of Shanghai Veterinary Research Institute, Chinese Academy of Agricultural Sciences (Shanghai, China; approval no: SV-20241129-Y01).

### Isolation and purification of *S. japonicum* eggs

*S. japonicum* eggs were extracted as described previously with some modifications (Zhu et al., 2016). Briefly, 8 weeks after infection with 1000 cercariae, the rabbit livers were ground into a homogenate using a high-speed tissue grinder. The homogenate was diluted with cold phosphate-buffered saline (PBS) containing 2% penicillin and streptomycin, and then filtered sequentially through 80-mesh and 100-mesh sieves. The filtered homogenate was transferred to a 50 mL centrifuge tube, centrifuged at 4°C, 300 × g for 5 min and the supernatant was discarded. Centrifugation was repeated several times until the supernatant was cleared. The liver homogenate was digested in 1 mg/mL collagenase IV (BioFroxx, Germany) and trypsin (Gibco) containing 0.02% EDTA for 2 h and 1 h, respectively. After washing with cold PBS, the eggs were resuspended in Dulbecco’s Modified Eagle Medium (DMEM) medium (Corning, USA) and the activity of the acquired eggs was detected with 0.4% Taipan blue stain. Eggs were thoroughly and gently washed three times with 20 mL PBS and then resuspended in preheated DMEM at 10^4^ eggs/mL.

### Establishment of a transwell model for LX-2 cells

In each transwell system of 12-well plate (PET membrane, pore size 0.4 μm) (Corning, USA), LX-2 cells (5×10^5^ cells/well) were placed in the lower well with 1.5 mL DMEM containing 10% FBS and 1% penicillin-streptomycin. Meanwhile, 10^4^ eggs were placed in the upper well with 900 μL fresh DMEM containing 10% FBS and 1% penicillin-streptomycin, designated as the Egg group. For the Control group, the upper chamber contained only the unused medium from *S. japonicum* egg preparations. Each transwell system had at least three technical replicates and were cultured in a humidified incubator at 37°C with 5% CO_2_. After 48 hours of incubation, cells were collected for subsequent RNA sequencing and isolation of cell surface proteins.

### Extraction of LX-2 cell surface proteins and MS analysis

After 48-hour co-incubation, cell surface proteins were isolated using the Pierce™ Cell Surface Biotinylation and Isolation Kit (Catalog Numbers: A44390, Thermo Fisher) following the manufacturer’s protocol. Cell lysates were prepared in buffer containing 4% SDS, 1 mM DTT, and 150 mM Tris-HCl (pH 8.0) with protease inhibitors, boiled for 3 min, sonicated on ice, reboiled, and clarified by centrifugation at 16,000 × g for 10 min at 25°C. Protein concentration was determined using the BCA assay (Bio-Rad, USA), and supernatants were stored at −80°C.

For protein digestion, 200 μg for each sample was performed according to the FASP procedure described by Wisniewski et al (Wiśniewski et al., 2009). Briefly, Samples were buffer-exchanged with UA buffer (8 M urea, 150 mM Tris-HCl, pH 8.0) using 10 kDa ultrafiltration units (Pall), followed by alkylation with 0.05 M iodoacetamide in UA buffer for 20 min in the dark. Filters were sequentially washed with UA buffer and 25 mM NH_4_HCO_3_. Proteins were digested overnight at 37°C with 3 μg trypsin (Promega) in 25 mM NH_4_HCO_3_. Peptides were collected by centrifugation.

Peptide concentrations were estimated by UV absorbance at 280 nm using an extinction coefficient of 1.1 for a 0.1% (g/L) solution. Equal amounts of iRT standard peptides were spiked into each sample. Peptides were desalted using C18 Empore™ SPE cartridges (Sigma), vacuum-dried, and reconstituted in 0.1% trifluoroacetic acid for MS analysis.

MS analysis was conducted on a timsTOF Pro instrument (Bruker) coupled with a NanoElute LC system (Bruker). Data were acquired in DIA mode using 54 windows, with MS/MS scans collected via 10 PASEF cycles. The instrument was operated in positive ion mode (scan range: 100–1700 m/z; 1/K_0_ range: 0.75–1.40 Vs/cm²; resolution: 30,000 at m/z 200). MS2 activation was achieved by HCD at a normalized collision energy of 30. Spectra were acquired in profile mode.

Raw MS data were analyzed using DIA-NN v1.8.1 against the *Homo sapiens* UniProtKB database (205,046 entries, downloaded 2024-10-23) and *Schistosoma japonicum* UniProtKB database (29,804 entries, downloaded 2025-01-09). Trypsin/P was specified as the cleavage enzyme, allowing up to two missed cleavages. Precursor and fragment mass tolerances were set at 6 ppm and 20 ppm, respectively. Carbamidomethylation of cysteines was set as a fixed modification; N-terminal acetylation and methionine oxidation were set as variable modifications. Peptide and protein identifications were filtered at a 1% FDR threshold.

To identify the membrane surface proteins of HSCs, the peptide fragments of proteins identified by MS from two groups were compared with both human and schistosome protein databases. Since some schistosome proteins share high similarity with their human homologs, schistosome-specific peptides were used to further screen these proteins, allowing for the distinction between schistosome-specific and non-schistosome-specific proteins.

### Bioinformatics analysis

To explore the biological functions of schistosome proteins, Protein-protein interaction (PPI) network, gene ontology (GO) and Kyoto Encyclopedia of Genes (KEGG) enrichment were analyzed for schistosome proteins identified in the Egg group using the STRING website (Version: 12.0) (Szklarczyk et al., 2023) and set the minimum required interaction score to 0.7 (high confidence) to increase the confidence of the prediction results. And proteins with no interaction were artificially removed from the analyzed results.

### RNA sequencing analysis

Total RNA was extracted from LX-2 cells by TRIzol reagent (Invitrogen, USA) according to the manufacturer’s instructions and quantified by Nanodrop (Thermo Scientific USA) (Rio et al., 2010). RNA samples are initially denatured at an appropriate temperature to disrupt their secondary structures, followed by mRNA enrichment using magnetic beads coated with oligo (dT). The reaction mixture is then prepared and incubated at the optimal temperature for a specified duration, after which the RNA is fragmented. The first-strand cDNA synthesis system is assembled, and the reaction is initiated to produce the first-strand cDNA. Subsequently, the second-strand cDNA synthesis system is prepared, and the reaction is carried out to generate double-stranded cDNA. Once the double-stranded cDNA is obtained, it undergoes end-repair, and a single ‘A’ nucleotide is appended to the 3’ ends of the blunt fragments. Adaptors are then ligated to the cDNA by configuring the appropriate reaction system and program. The resulting product is amplified through PCR. The PCR products are denatured to yield single-stranded DNA, which is then circularized by setting up the corresponding reaction system and program. Linear DNA molecules that fail to circularize are removed, leaving only single-stranded circular DNA. These circular DNA molecules are amplified via rolling circle amplification, producing DNA nanoballs (DNBs) containing multiple copies of the DNA. High-quality DNBs are subsequently loaded onto patterned nanoarrays using advanced DNA nanochip technology and sequenced via combinatorial Probe-Anchor Synthesis (cPAS).

The raw data was filtered with SOAPnuke (v1.6.5) (Chen et al., 2018) by (1) Removing reads containing adapters (adapter contamination); (2) Removing reads whose unknown base (‘N’ base) ratio is more than 1%; (3) Removing reads whose low-quality base ratio (Base quality less than or equal to 15) is more than 40%, afterwards clean reads were obtained and stored in FASTQ format. The subsequent analysis and data mining were performed on Dr. Tom Multi-omics Data mining system (https://biosys.bgi.com).

The clean data were mapped to the reference genome (*Homo sapiens*: GCF_000001405.39_GRCh38.p13) by HISAT (v2.2.1) (Kim et al., 2015). The clean data were mapped to the assembled unique gene by Bowtie2 (v2.4.5) (Langmead and Salzberg, 2012). The expression level of genes was calculated by RSEM (v1.3.1) (Li and Dewey, 2011). Time Series analysis was performed by Mfuzz (v2.60.0) (Kumar and Futschik, 2007), and gene co-expression network analysis was performed by WGCNA (v1.71). Analysis of differentially expressed genes (DEGs) was performed using the DEseq2 (Love et al., 2014)/DEGSeq (Wang et al., 2010) under the conditions of |Log2Fold Change| ≥ 1 and adjusted *P* value ≤ 0.05 for analysis of DEGs.

### Bioinformatics analysis of DEGs

To analyze the function of the DEGs, the GO and KEGG annotation were performed. KEGG enrichment analysis was performed using the phyper function in R, while GO enrichment analysis utilized the TermFinder package. The candidate DEGs with a Q value of ≤ 0.05 were defined as significantly enriched.

### Real-time quantitative polymerase chain reaction

To verify the accuracy of RNA sequencing results, RNA sequencing samples were performed for RT-qPCR. The reverse-transcription was performed using a Hifair III 1st Strand cDNA Synthesis SuperMix for qPCR kit (Yeasen, China). The resulting cDNA was used as template for qPCR with Hieff qPCR SYBR Green Master Mix (Yeasen, China). The relative mRNA expression levels of genes were quantified with β-actin served as an endogenous control. The LightCycler 96 system (Roche, China) was used for qPCR analysis. The cycling conditions were as follows: preincubation, 95°C for 60 s; 2 step amplification, 95°C for 5 s, and 60°C for 30 s, for 40 cycles; melting, 95°C for 10 s, 65°C for 60 s, 97°C for 1 s. All samples were assessed in triplicate. The 2^-ΔΔCt^ method (Livak and Schmittgen, 2001) was used to calculate the fold change in the expression of all the mRNAs and all samples were assessed in triplicate. The primers used in this study are listed in **Supplementary Table S1**.

### Statistical analysis

The data were analyzed with the Student’s t-test and are expressed as the mean ± S.D. of three independent biological replicates. A *P* value of <0.05 was considered statistically significant.

## Results

### Identification of membrane surface proteins of HSCs and bioinformatics analysis

MS analysis was performed on peptides derived from enzymatic digestion to profile the membrane surface proteins of HSCs from the Egg and Control groups. Peptides identified from each group were compared against human and schistosome protein databases. A total of 88 schistosome-derived proteins were identified in the Egg group (**Table 1**). Due to the high sequence similarity between certain schistosome proteins and their human homologs, further screening was performed using schistosome-specific peptides. This additional analysis identified 36 schistosome-specific peptides, corresponding to 34 unique schistosome-specific proteins.

**Figure 1:**
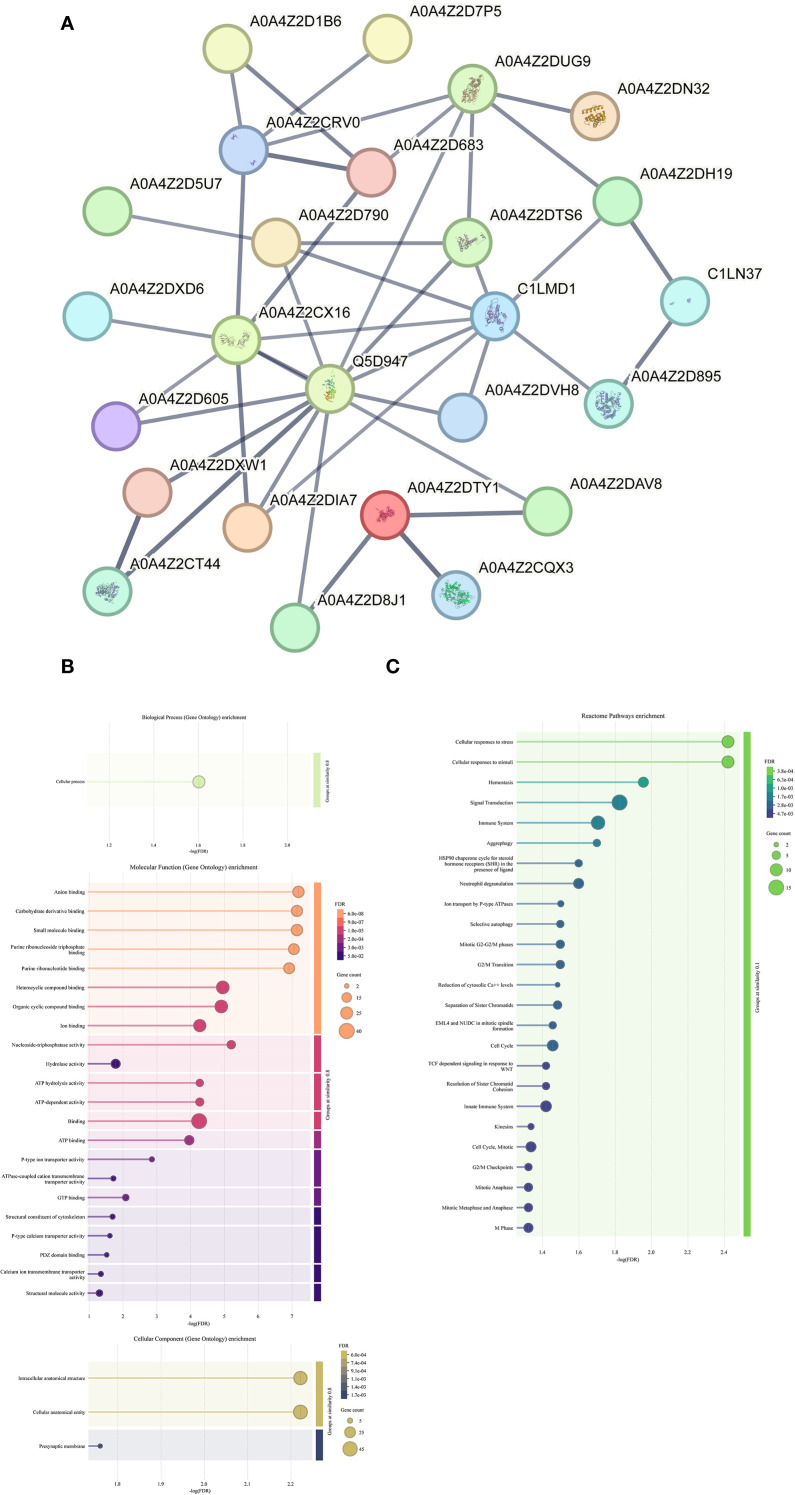
Identification and bioinformatics analysis of hepatic stellate cell membrane surface proteins. (**A**) The protein-protein interaction network of 88 schistosomal proteins. The minimum required interaction score is high confidence (0.700). The meaning of the network edges: confidence (line thickness indicates the strength of data support). **(B)** GO enrichment analysis of 88 schistosomal proteins. **(C)** KEGG enrichment analysis of 88 schistosomal proteins.

Subsequently, bioinformatic analysis were conducted on these 88 proteins. PPI network analysis indicated that key hub proteins—such as Histone H3 (A0A4Z2CRV0), Actin-1 (A0A4Z2DUG9), Heat shock protein HSP 90-alpha (Q5D947), Cell division control protein 42 (C1LMD1), and Polyubiquitin (A0A4Z2CX16)—exhibited multiple interaction nodes (**Figure 1A**). GO enrichment analysis indicated that these proteins were predominantly involved in biological processes (BP) related to cellular processes. In terms of molecular function (MF), enriched terms included anion binding, carbohydrate derivative binding, and small molecule binding. For cellular component (CC) annotation, proteins were enriched mainly at presynaptic membranes, intracellular anatomical structures, and cellular anatomical entities. Reactome pathway enrichment analysis further revealed involvement of these proteins in 25 pathways, notably including cellular responses to stress, responses to stimuli, and hemostasis (**Supplementary Table S2**). A similar analysis was attempted for the subset of 34 schistosome-specific proteins; however, due to the limited number, meaningful bioinformatic insights could not be obtained.

### Transcriptome sequencing reveals differential gene expression profiles of HSCs co-cultured with schistosome eggs

**Figure 2:**
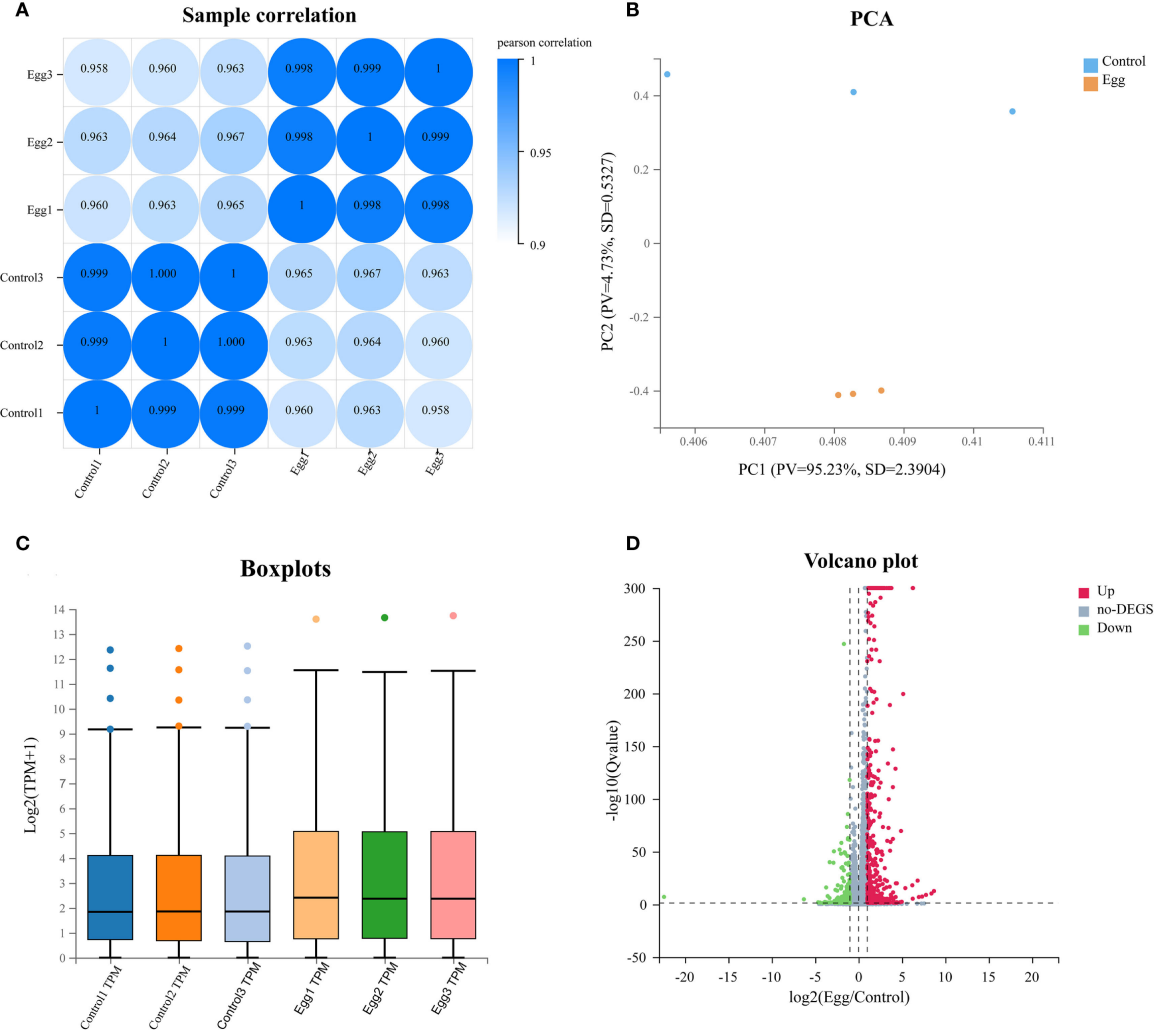
Sequencing quality control and DEGs expression profiling. **(A)** Samples correlation heatmap of Control and Egg groups. **(B)** Principal component analysis (PCA) plot of Control and Egg groups. **(C)** Boxplot of gene expression levels between Control and Egg groups. **(D)** Volcano plot of differentially expressed genes between Control and Egg groups.

To investigate whether schistosome eggs placed in the upper chamber of a Transwell system remotely influence hepatic stellate cells (HSCs) cultured in the lower chamber, transcriptome sequencing was conducted on HSC samples from the Egg and Control groups. Pearson correlation analysis among biological replicates indicated high intra-group correlation coefficients and low variability, confirming strong consistency among replicate samples (**Figure 2A**). Principal component analysis (PCA) further demonstrated clear separation between Egg and Control groups, highlighting distinct global gene expression patterns and suggesting that the transcriptional profiles of HSCs were significantly altered by co-culture with schistosome eggs (**Figure 2B**). Box plot analysis additionally confirmed similar expression distributions within each group, indicating good parallelism and reproducibility across all samples (**Figure 2C**). A volcano plot was employed to visualize the distribution of differentially expressed genes (DEGs), resulting in the identification of 454 significantly upregulated genes and 180 significantly downregulated genes in the Egg group compared with the Control group (**Figure 2D**, **Supplementary Table S3**).

**Figure 3:**
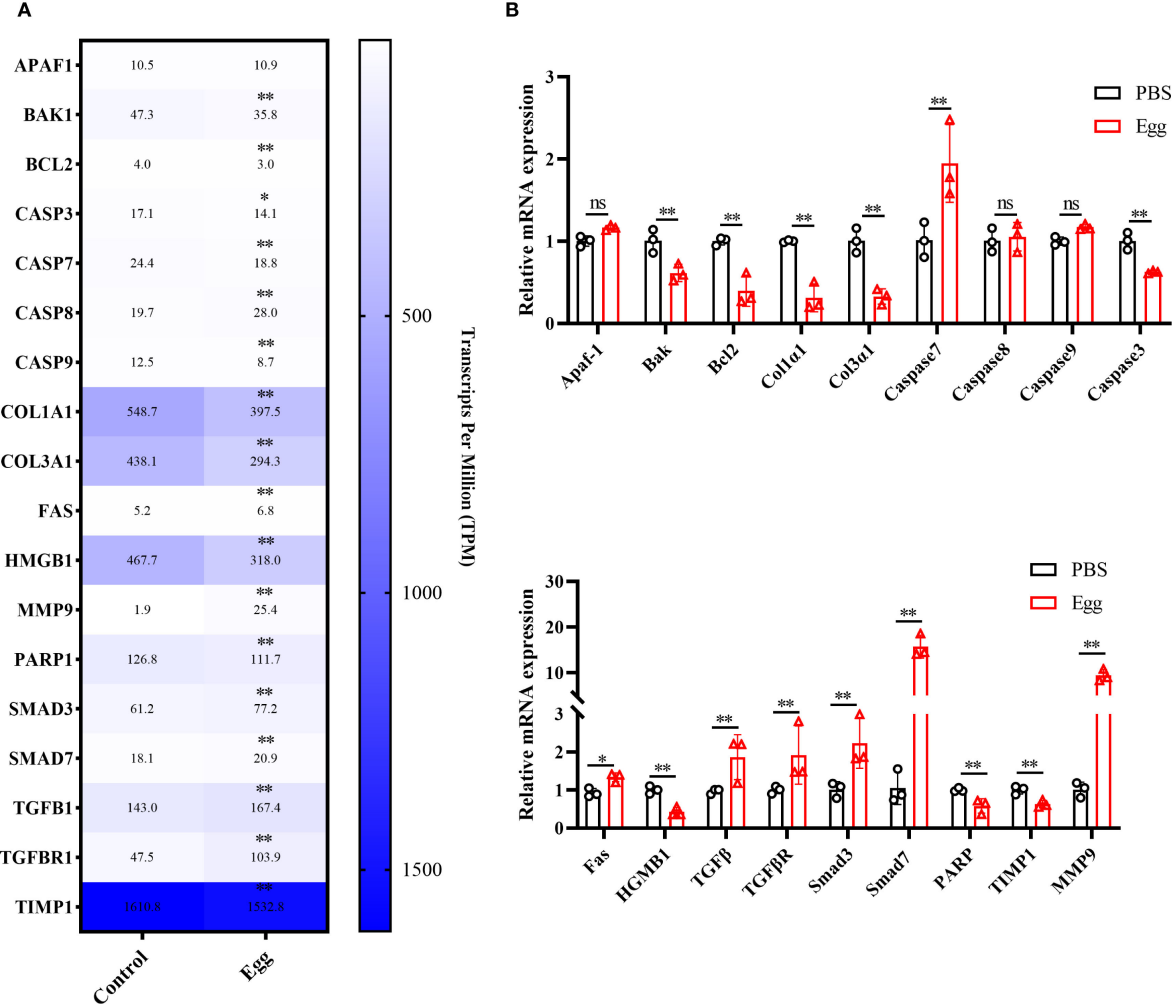
RT-qPCR validation. **(A)** Heatmap of the expression levels of 20 DEGs. **(B)** RT-qPCR validation. Results are shown as mean ± SD (one-way ANOVA Dunnett’s multiple comparison test).

To validate the accuracy of RNA-sequencing data, 18 randomly selected DEGs underwent further analysis by RT-qPCR (**Figure 3**). Expression patterns of 15 genes were consistent between RNA-seq and RT-qPCR results, confirming the reliability of the sequencing data.

### Functional enrichment analysis of DEGs

To further investigate the biological roles and metabolic pathways associated with the 634 DEGs, GO and KEGG enrichment analyses were separately conducted on the 454 upregulated and 180 downregulated genes. The top 40 significantly enriched terms for each category were visualized and analyzed.

GO enrichment analysis of the upregulated DEGs (**Supplementary Figure S1A–C**) revealed that in the BP category, genes were predominantly involved in inflammatory responses, extracellular matrix organization, and positive regulation of cell migration. In the MF category, significant enrichment was observed for cytokine activity, collagen binding, and transmembrane signaling receptor activity. Regarding CC, the upregulated DEGs were primarily annotated to plasma membrane, collagen-containing extracellular matrix, and extracellular exosomes. KEGG pathway analysis (**Supplementary Figure S1D**) further demonstrated significant enrichment in inflammation- and fibrosis-related signaling pathways, notably cytokine-cytokine receptor interaction, IL-17 signaling, and Toll-like receptor signaling pathways. Additionally, enrichment was observed in pathways involved in apoptosis and cell cycle regulation, including the PI3K-Akt signaling pathway, apoptosis, and MAPK signaling pathway.

For downregulated DEGs, GO enrichment analysis (**Supplementary Figure S2A–C**) indicated predominant involvement in biological processes related to cell adhesion, positive regulation of macrophage chemotaxis, and regulation of muscle filament sliding speed. MF analysis highlighted enrichment of microfilament motor activity and extracellular matrix structural constituents conferring tensile strength. Within the CC category, significant annotations included extracellular space, plasma membrane, and collagen-containing extracellular matrix. KEGG pathway analysis (**Supplementary Figure S2D**) further indicated significant enrichment in the Hippo signaling pathway, Hepatitis B pathway, and AMPK signaling pathway. Collectively, these enrichment analyses demonstrate that the identified DEGs are closely involved in regulating diverse biological functions and signaling pathways in HSCs, suggesting their potential roles in modulating cellular responses during liver fibrosis.

## Discussion

The deposition of *S. japonicum* eggs in hepatic tissue is a recognized key factor in the development of schistosome-induced hepatic fibrosis (Liu et al., 2022). While activation of HSCs is recognized as central to fibrotic progression, a process marked by dysregulated extracellular matrix (ECM) remodeling, the precise mechanisms by which schistosome eggs modulate HSCs activity remain incompletely characterized. Egg-derived ESPs, consisting of diverse proteins, glycans, and lipids, have been demonstrated to induce fibrosis and severe inflammatory responses in the host liver (Carson et al., 2020). To investigate the modulation of HSCs by *S. japonicum* eggs, a transwell co-culture system was employed.

Proteomic profiling of HSC surface proteins following co-culture identified 88 schistosome proteins (**Table 1**). Notably, several key proteins, identified as hub components of the PPI network, were filtered. Among these, the *S. mansoni* Actin-1 promoter, which upregulates the expression of the schistosomulum Caspase 7 gene and induces worm death (Liang et al., 2014), may also regulate similar processes in HSCs. Indeed, our study observed upregulation of Caspase 7 in HSCs following co-culture with schistosome eggs, suggesting that *S. japonicum* Actin-1 may regulate Caspase 7 expression through a homologous mechanism, potentially contributing to HSC apoptosis.

Heat shock protein HSP 90-alpha, a well-known marker protein of EVs, is enriched between the egg shell and miracidia (Xu et al., 2020). It may mediate interactions between schistosome eggs and HSCs via EVs, further promoting fibrosis. Additionally, Cell division control protein 42 (CDC42), a member of the Rho GTPase family, is significantly upregulated in the host during the progression of non-alcoholic fatty liver disease and is closely associated with gene networks involved in inflammation, fibrosis, and hepatocellular carcinoma (Hong et al., 2022).

Polyubiquitin in schistosomes remains poorly studied, but research indicates that the host polyubiquitin pathway is tightly linked to the progression of liver cancer. Specifically, K48-linked polyubiquitination plays a critical role in hepatocellular carcinoma by promoting the degradation of c-Myc and enhancing glucose metabolism reprogramming in tumor cells (Xia et al., 2023). The conserved nature of polyubiquitin and other key proteins across the host and schistosome suggests they may influence the transcriptional and translational regulation of DEGs in HSCs via ESPs released by schistosome eggs.

Further filtering of the identified proteins using schistosome-specific peptides yielded 34 *S. japonicum*-specific proteins (**Table 1**), including Heat shock protein 70 (A0A0N7I6P5), Filamin-A isoform 1 (A0A4Z2CZ15), Basigin (A0A4Z2DHH0), and sodium/potassium-transporting ATPase subunit alpha-1 (A0A4Z2CUM2), which overlapped with previously reported schistosome EV proteins (Du et al., 2020; Zhong et al., 2024). These proteins, particularly filamin-A (FLNA) and basigin (CD147), emerged as compelling candidates. FLNA, a cytoskeletal regulator, is upregulated during HSC activation and fibrosis progression (Zhang et al., 2017; Zhao et al., 2023). CD147, known to participate in ECM remodeling and fibrosis regulation (Guindolet and Gabison, 2020), promotes TGF-β1 synthesis via the ERK1/2-Sp1 signaling cascade, further enhancing HSC activation (Li et al., 2015). Similarly, ATP1A1, another protein identified in our dataset, has been associated with fibrotic progression by promoting cell migration and proliferation, particularly in hepatocellular carcinoma (Darce et al., 2023). These findings collectively suggest that egg-secreted proteins may promote fibrogenesis by upregulating mediators like MMP9 and TGF-β1, though functional validation is warranted.

Intriguingly, our data also revealed anti-fibrotic effects. Sjp40 and Sjp70, which have previously shown to downregulate α-SMA and COL1A1 in HSCs (Xu et al., 2020), were detected in the egg proteome. Transcriptomic analysis further identified downregulation of fibrosis-associated genes, implying a dual regulatory role for schistosome eggs in balancing pro- and anti-fibrotic pathways (Duan et al., 2014; Li et al., 2025). Due to the inherent sequence similarity between parasite-derived proteins and host homologs, identification of proteins in this study relied heavily on database alignment strategies, carrying a risk of false-positive identification. This constitutes an acknowledged limitation of the current homology-based proteomic approach.

In addition, transcriptomic analysis reinforced these observations: upregulated DEGs were enriched in inflammation, cancer, and fibrosis pathways, a result that is consistent with the known association between advanced schistosomiasis and hepatocellular carcinoma (Darce et al., 2023) Downregulated DEGs, though fewer, implicated steroid hormone biosynthesis, aligning with reports that glucocorticoids suppress macrophage infiltration (Petrescu et al., 2017) and HSC activation via TGF-β inhibition (Bolkenius et al., 2004).

In summary, this study demonstrates that *S. japonicum* eggs remotely regulate HSC transcriptional programs via secreted factors, unveiling potential therapeutic targets for mitigating hepatic fibrosis.

## Data Availability

Sequencing data for RNA-seq have been deposited to Sequence Read Archive at the National Center for Biotechnical Information under accession number CNP0007747.

## Supplementary material

Supplementary Figure 1 GO and KEGG enrichment analysis of upregulated DEGs. (A) GO enrichment analysis (biological process) Top 40. (B) GO enrichment analysis (molecular function) Top 40. (C) GO enrichment analysis (cellular components) Top 40. Red underlining denotes terms associated with collagen production, blue underlining denotes terms related to inflammation, and green underlining denotes terms related to cell activity. (D) KEGG enrichment analysis Top 40. The bubble size indicates the number of DEGs annotated to a specific GO term, while the color represents the enrichment significance, as indicated by the Qvalue.

Supplementary Figure 2 GO and KEGG enrichment analysis of downregulated DEGs. (A) GO enrichment analysis (biological process) Top 40. (B) GO enrichment analysis (molecular function) Top 40. (C) GO enrichment analysis (cellular components) Top 40. Red underlining denotes terms associated with collagen production, blue underlining denotes terms related to inflammation, and green underlining denotes terms related to cell activity. (D) KEGG enrichment analysis Top 40. The bubble size indicates the number of DEGs annotated to a specific GO term, while the color represents the enrichment significance, as indicated by the Qvalue.
