# Supplementary figures and images for: Membrane proteomics and transcriptomic profiling analysis of hepatic stellate cells co-incubated with Schistosoma japonicum eggs
Source: Front Cell Infect Microbiol. 2025 Sep 16;15:1674880. doi: 10.3389/fcimb.2025.1674880 (PMC12479499; doi:10.3389/fcimb.2025.1674880)

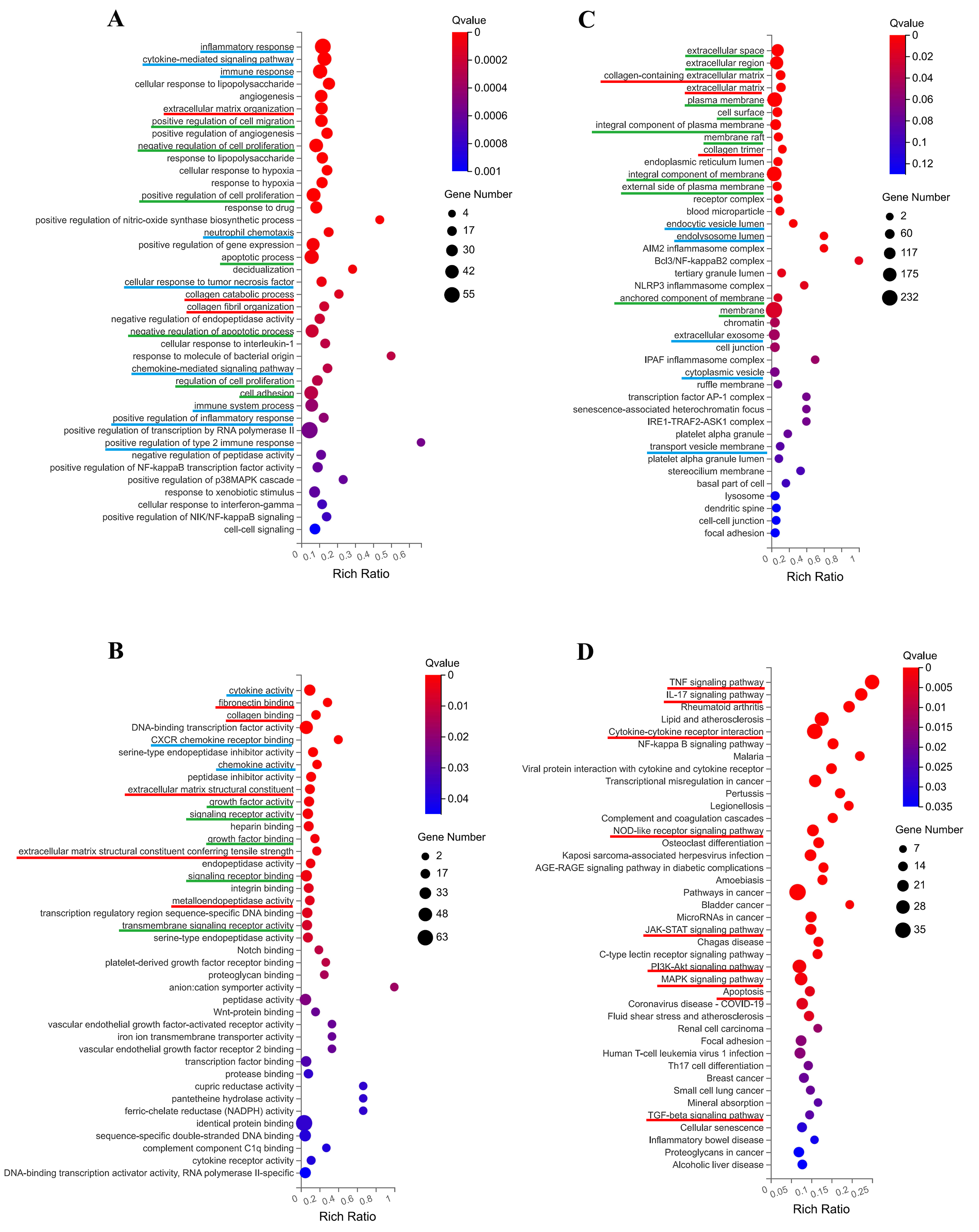

Supplement: Supplementary Figure 1 — GO and KEGG enrichment analysis of upregulated DEGs. (A) GO enrichment analysis (biological process) Top 40. (B) GO enrichment analysis (molecular function) Top 40. (C) GO enrichment analysis (cellular components) Top 40. Red underlining denotes terms associated with collagen production, blue underlining denotes terms related to inflammation, and green underlining denotes terms related to cell activity. (D) KEGG enrichment analysis Top 40. The bubble size indicates the number of DEGs annotated to a specific GO term, while the color represents the enrichment significance, as indicated by the Qvalue. [file Image1.tif]

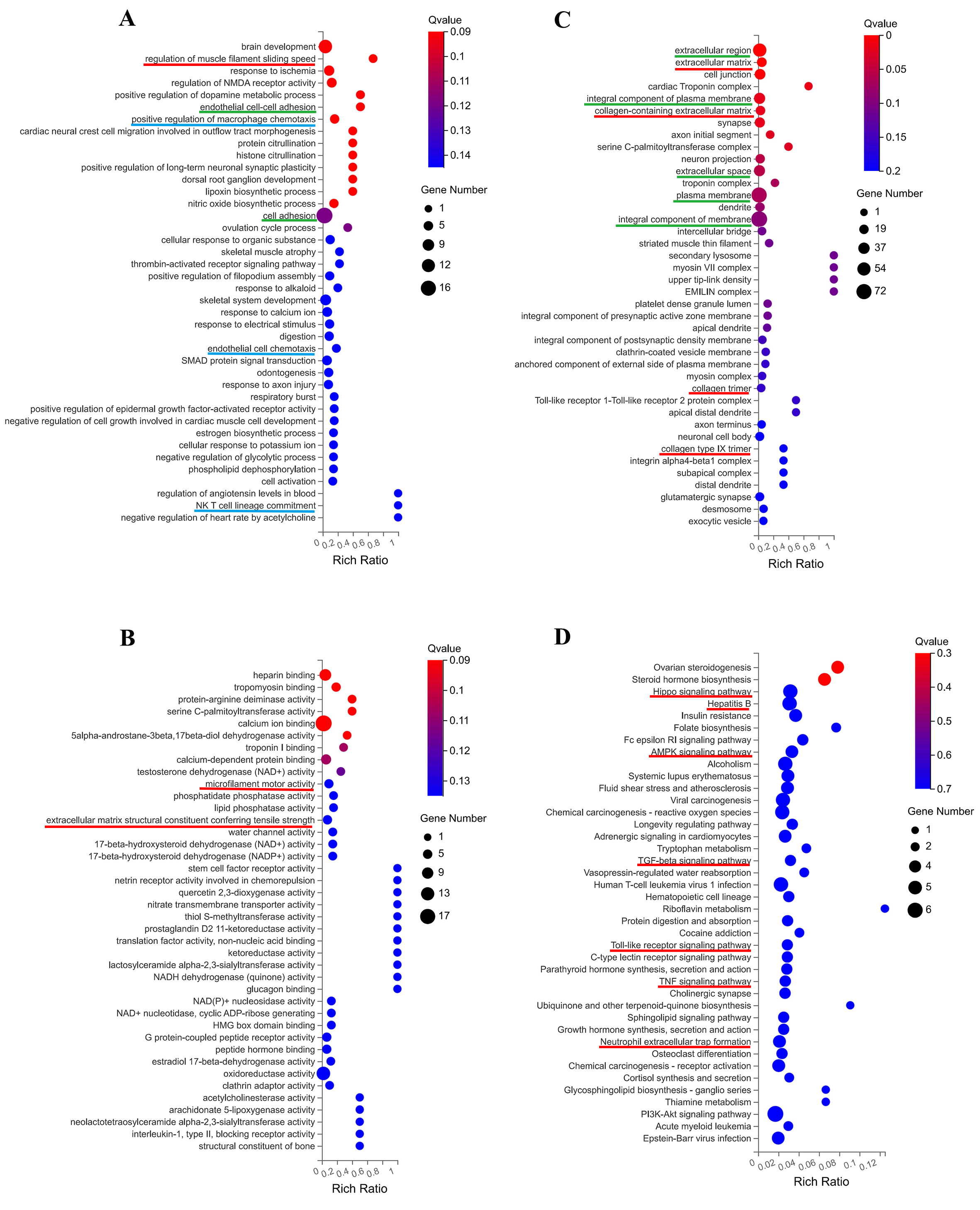

Supplement: Supplementary Figure 2 — GO and KEGG enrichment analysis of downregulated DEGs. (A) GO enrichment analysis (biological process) Top 40. (B) GO enrichment analysis (molecular function) Top 40. (C) GO enrichment analysis (cellular components) Top 40. Red underlining denotes terms associated with collagen production, blue underlining denotes terms related to inflammation, and green underlining denotes terms related to cell activity. (D) KEGG enrichment analysis Top 40. The bubble size indicates the number of DEGs annotated to a specific GO term, while the color represents the enrichment significance, as indicated by the Qvalue. [file Image2.tif]
