# Supplementary material for: Membrane proteomics and transcriptomic profiling analysis of hepatic stellate cells co-incubated with Schistosoma japonicum eggs
Source: Front Cell Infect Microbiol. 2025 Sep 16;15:1674880. doi: 10.3389/fcimb.2025.1674880 (PMC12479499; doi:10.3389/fcimb.2025.1674880)
Supplement: Supplementary file 3 [file Table1.docx]

S1 Table. Primers used in the experiment

| **Primer** | **Sequences (5’-3’)** |
| --- | --- |
| Human β-actin-F | GATGTGGATCAGCAAGCAGGAG |
| Human β-actin-R | AGAAAGGGTGTAACGCAACTAAGTC |
| Human Apaf-1-F | GCCTCAGCCTCCCAAGTAGC |
| Human Apaf-1-R | CAAGACCAGCCTAGACAACATAGTG |
| Human α-SMA-F | CGTGGCTATTCCTTCGTTACTACTG |
| Human α-SMA-R | CCATCAGGCAACTCGTAACTCTTC |
| Human Bak-F | GACGACATCAACCGACGCTATG |
| Human Bak-R | GCTGGTGGCAATCTTGGTGAAG |
| Human Bax-F | CCAAGAAGCTGAGCGAGTGTC |
| Human Bax-R | GTCCACGGCGGCAATCATC |
| Human Bcl-2-F | TTGTTGTTGTTCAAACGGGATTCAC |
| Human Bcl-2-R | GCAGCCAGCCAGCAATTAGC |
| Human Caspase9-F | ATTTGGTGATGTCGGTGCTCTTG |
| Human Caspase9-R | CACGGCAGAAGTTCACATTGTTG |
| Human Caspase7-F | GTCTCACCTATCCTGCCCTCAC |
| Human Caspase7-R | TGTTCTTCTCCTGCCTCACTGTC |
| Human Caspase8-F | TTTGACCACGACCTTTGAAGAGC |
| Human Caspase8-R | GAGGATACAGCAGATGAAGCAGTC |
| Human Caspase3-F | GCTGCCTGTAACTTGAGAGTAGATG |
| Human Caspase3-R | GCGTATGGAGAAATGGGCTGTAG |
| Human-Col1α1-F | GGTTCGGAGGAGAGTCAGGAAG |
| Human-Col1α1-R | TTTCAGCAACACAGTTACACAAGG |
| Human-Col3α1-F | TGGTCTGCAAGGAATGCCTGGA |
| Human-Col3α1-R | TCTTTCCCTGGGACACCATCAG |
| Human Fas-F | ACTGCGTGCCCTGCCAAG |
| Human Fas-R | CCACTTCTAAGCCATGTCCTTCATC |
| Human TGFB1-F | AAGGTGAGGAAACAAGCCCAGAG |
| Human TGFB1-R | AAGTGCTAGGATTACAGGCGTGAG |
| Human TGFBR-F | CTGCCTGTCGGTGAGATTGGTTC |
| Human TGFBR-R | GGACCAGCAAGCAGGAGAGC |
| Human-Smad3-F | GGAGCGGAGTACAGGAGACAGAC |
| Human-Smad3-R | CTAAGACACACTGGAACAGCGGATG |
| Human-Smad7-F | CTCGGAAGTCAAGAGGCTGTGTTG |
| Human-Smad7-R | TCTAGTTCGCAGAGTCGGCTAAGG |
| Human-PARP-F | CAGAGTATGCCAAGTCCAACAGAAG |
| Human-PARP-R | CAGCGGTCAATCATGCCTAGC |
| Human MMP9-F | CCCTGGTCCTGGTGCTCCTG |
| Human MMP9-R | CTGCCTGTCGGTGAGATTGGTTC |
| Human TIMP1-F | TGGCTTCTGGCATCCTGTTGTTG |
| Human TIMP1-R | CCTGATGACGAGGTCGGAATTGC |
