# Supplementary material for: Membrane proteomics and transcriptomic profiling analysis of hepatic stellate cells co-incubated with Schistosoma japonicum eggs
Source: Front Cell Infect Microbiol. 2025 Sep 16;15:1674880. doi: 10.3389/fcimb.2025.1674880 (PMC12479499; doi:10.3389/fcimb.2025.1674880)
Supplement: Supplementary file 6 [file Table4.docx]

Table 1 MS results for 88 schistosome proteins of the Egg group

| **Schistosome-specific proteins (34/88)** | | | | | | | |
| --- | --- | --- | --- | --- | --- | --- | --- |
|  |  | **Egg group** | | | **Control group** | | |
| Protein Accessions | Protein Descriptions | Slot1-7_1_8098.d.  PG.Quantity | Slot1-7_1_8099.d.  PG.Quantity | Slot1-7_1_8100.d.PG.Quantity | Slot1-8_1_8101.d.PG.Quantity | Slot1-8_1_8102.d.PG.Quantity | Slot1-8_1_8103.d.  PG.Quantity |
| A0A4Z2DHH0 | Basigin | — | 632.8504638671875 | 488.73992919921875 | 8096.58349609375 | 8774.521484 | 10473.873046875 |
| A0A4Z2CRP6 | Suppressor of lurcher protein | 1056.9449462890625 | 1671.39501953125 | 1838.53857421875 | — | — | — |
| C1LE79 | Calcium-transporting ATPase | 565.2234497070312 | 619.4214477539062 | 603.42822265625 | 4360.31494140625 | 892.126708984375 | 2567.80517578125 |
| A0A4Z2CUJ3 | Coiled-coil domain-containing protein 86 | 226.03387451171875 | 167.390869140625 | 156.9310760498047 | 5587.72314453125 | 4954.7099609375 | 4658.54541015625 |
| A0A0N7I6P5 | Heat shock protein 70 | 5244.3037109375 | 5579.783203 | 5477.68408203125 | — | — | — |
| A0A4Z2D1B6 | Protein arginine N-methyltransferase 3 | 98.2738037109375 | — | 73.68830108642578 | 579.7907104492188 | 301.8111877441406 | 448.4101867675781 |
| A0A4Z2D270 | tRNA-dihydrouridine(16/17) synthase [NAD(P)(+)] | 898.6263427734375 | — | — | 1890.5244140625 | 2209.129150390625 | 1849.448974609375 |
| A0A4Z2D605 | Glycogen synthase kinase-3 alpha | 81.29642486572266 | — | 69.5511245727539 | 360.8625183105469 | 364.2896728515625 | 360.58819580078125 |
| A0A4Z2D790 | small monomeric GTPase | — | — | 1018.6177368164062 | 2487.645751953125 | 177.21490478515625 | 1263.4085693359375 |
| A0A4Z2D7P5 | Forkhead box protein I2 | 152.97021484375 | — | — | 2532.43115234375 | — | — |
| A0A4Z2D859 | Ecotropic viral integration site 5 protein | 779.7864379882812 | — | 353.3582763671875 | 36.111427307128906 | 42.25910186767578 | 44.188716888427734 |
| A0A4Z2CZ15 | Filamin-A isoform 1 | 387.5325622558594 | 452.8447570800781 | 466.8797912597656 | 374.2772216796875 | 431.8038635253906 | 333.27911376953125 |
| A0A4Z2D8J1 | peptidylprolyl isomerase | 2387.809326171875 | 3652.02392578125 | 3299.145751953125 | 272.0032958984375 | 382.2056579589844 | 655.8818359 |
| A0A4Z2DFX6 | Nucleobindin-1 isoform 2 | — | 826.430908203125 | 892.7122802734375 | 820.2540283203125 | 628.065673828125 | 1065.818359 |
| A0A4Z2DL59 | Gluconokinase | 145.31912231445312 | — | — | — | 3055.52783203125 | — |
| A0A4Z2DLE1 | DNA replication licensing factor MCM6 | 142.3269805908203 | 199.61590576171875 | — | 486.974365234375 | 341.8231201171875 | 477.2324219 |
| A0A4Z2CUM2 | Sodium/potassium-transporting ATPase subunit alpha | 1343.54541 | 1712.185181 | 1538.441406 | 90.77145386 | 99.04307556 | 103.1371994 |
| A0A4Z2DNK3 | Anosmin-1 | — | 342.9745483 | 351.156311 | 2367.7771 | 711.0629272 | 1344.344116 |
| A0A4Z2DPZ4 | Gsx family homeobox | 1034.93396 | 1516.612549 | 1327.268433 | 1767.742676 | 764.5385742 | 958.1546631 |
| A0A4Z2DTW2 | Tetratricopeptide repeat protein | 166.9579468 | — | 214.9068756 | 84.87389374 | 33.8850708 | 82.53833008 |
| A0A4Z2DXD6 | Eukaryotic translation initiation factor 4 gamma 2 | 159.156601 | 57.43087769 | 160.5142365 | 430.5010071 | 357.9969177 | 412.5149841 |
| A0A4Z2DYB8 | Transient receptor potential cation channel subfamily M member 3 | — | — | 2417.688477 | 365.3057861 | 259.6915894 | 295.8439636 |
| C1L995 | Putative tyrosine 3-monooxygenase | 3256.212646 | 1512.609497 | 2679.670654 | 3011.466309 | 2754.609131 | 4358.734375 |
| C1LG11 | Small ribosomal subunit protein mS40 | 124.636673 | 134.9674835 | 182.4095459 | — | 1190.309082 | 680.9490967 |
| C1LGA0 | Egg protein CP111 | 3324.988525 | — | 1750.654053 | 31784.08594 | 25037.10938 | 28052.12695 |
| C1LL94 | Plasmodium RESA N-terminal domain-containing protein | 692.1602783 | 783.8122559 | 967.2662354 | 4459.476074 | 4413.056152 | 6261.369629 |
| C1LXY2 | UPF0506 domain-containing protein | — | — | 185.2317963 | 64.83396912 | 63.16259003 | 46.65431213 |
| C7TZT1 | DnaJ homolog, subfamily B, member 4 | 61.23542023 | — | 47.75883865 | 139.65802 | 143.5727692 | 146.451889 |
| Q5BXB8 | SJCHGC08236 protein (Fragment) | — | — | 886.746582 | 1002.746887 | 790.5075073 | 1695.91748 |
| Q5C2Q3 | Uncharacterized protein | 281.0413818 | 263.2141724 | 311.5569458 | — | 450.8084106 | 417.5675659 |
| A0A4Z2DFM9 | Exportin-1 isoform 2 | 907.7197266 | 593.4896851 | — | 1273.752441 | 1267.863403 | 1299.790894 |
| Q5DCA1 | 6-pyruvoyltetrahydropterin synthase | 521.5338135 | — | — | 250.4281464 | 252.5644073 | 253.3547974 |
| Q5DHR6 | SJCHGC03305 protein | 73.10479736 | 84.69926453 | — | 4878.274414 | 18347.92969 | 12246.43555 |
| Q5DHW4 | EF-hand domain-containing protein | 1597.872437 | 1749.669556 | 2036.194458 | 159.2685089 | 134.8833008 | 464.507019 |
| **Schistosome non-specific proteins (54/88)** | | | | | | | |
| A0A289ZET2 | RNA helicase | — | — | 60.44192886 | — | — | — |
| A0A4Z2CPE1 | Pyruvate kinase | 503.4674987792969 | 981.00354 | 2136.164307 | — | — | — |
| A0A4Z2CQE2 | Pumilio 1 isoform 3 | 57.96179962158203 | — | — | — | — | — |
| A0A4Z2CQE8 | Mitochondrial-processing peptidase subunit beta isoform 2 | 105.29412841796875 | — | — | — | — | — |
| A0A4Z2CQX3 | RNA helicase | 100.9334945678711 | 257.5843201 | 171.1005859 | — | — | — |
| A0A4Z2CRQ5 | Large ribosomal subunit protein eL24 | 538.7692261 | 620.4597778 | 599.223877 | — | — | — |
| A0A4Z2CRV0 | Histone H3 | 223.5775146 | 146.4030457 | 159.2915039 | — | — | — |
| A0A4Z2D895 | Tubulin alpha-3 chain | 7313.352539 | 7952.73877 | 7014.808105 | 191.5061646 | 242.5326996 | 307.1883545 |
| A0A4Z2CT44 | Stress-70 protein isoform 1 | 524.4136353 | 485.7735596 | 474.9728088 | — | — | — |
| A0A4Z2CV62 | Large ribosomal subunit protein uL16 | 468.0605469 | 281.3656006 | 546.5665894 | 75.93847656 | 93.88043976 | 75.40483856 |
| A0A4Z2CWY6 | Band 4.1 domain-containing protein | 257.4812317 | 312.9268799 | 224.4187775 | — | 40.66808319 | — |
| A0A4Z2CX16 | Polyubiquitin | 4727.999023 | 2634.799805 | 2994.678223 | 240.4296722 | 214.715332 | 229.1774597 |
| A0A4Z2CYV9 | 2-oxoglutarate dehydrogenase, mitochondrial | 206.0658722 | 230.4959412 | 187.9940186 | 986.6287842 | — | 707.828186 |
| A0A4Z2DN32 | Alpha-actinin | 46.76910782 | 60.78980637 | 64.94520569 | 89.66517639 | — | — |
| A0A4Z2CZN8 | DNA replication licensing factor MCM2 | 604.7457886 | 524.3723145 | 497.5179749 | 170.0702362 | 197.0228729 | 118.8285446 |
| A0A4Z2D076 | ATPase family AAA domain-containing protein isoform 2 | 1804.336426 | 1716.649048 | 1399.921875 | 461.1947327 | 417.7130737 | 441.5752869 |
| A0A4Z2D2W8 | Kinesin-like protein unc-104 | 33.6830864 | 10.36001015 | 60.14898682 | 214.049881 | 241.7634277 | 235.3122559 |
| Q5D947 | Heat shock protein HSP 90-alpha | 4347.985352 | 7116.858398 | 6579.821289 | 328.1893921 | 412.3291321 | 53.08579254 |
| A0A4Z2D4A8 | Elongation factor 1-alpha | 670.2369385 | 1149.779419 | 669.954834 | 8141.521973 | 10567.8877 | 12583.73926 |
| A0A4Z2D4P7 | 26S proteasome regulatory subunit 6A isoform 2 | 32.43699265 | — | — | 229.7392883 | 214.5797577 | 224.3918762 |
| A0A4Z2D5U7 | 14-3-3 protein 1 | 79.13332367 | 107.1681747 | 99.39880371 | 414.1003418 | 389.5269775 | 559.005127 |
| A0A4Z2D662 | Splicing factor 3B subunit 1 isoform 1 | 206.8077545 | 200.138916 | 209.2781067 | 1169.949829 | 1253.457886 | 1388.343018 |
| A0A4Z2D683 | Histone H4 | 1503.034424 | 1912.419556 | 1731.808716 | 241.9254456 | 145.1702728 | 143.9157562 |
| A0A4Z2DXY6 | ATP synthase subunit alpha | 283.7767944 | 267.0193787 | 301.3030396 | 119.8920975 | 124.8952255 | 132.9813995 |
| A0A4Z2DB62 | Myosin-11 isoform 2 | 718.522644 | 966.1309814 | 441.9997864 | 142.8782501 | 131.2624969 | 128.7054443 |
| A0A4Z2DF64 | Plasma membrane calcium-transporting ATPase 2 isoform 1 | 1234.132202 | 472.868042 | 791.0932007 | 306.9606628 | 200.138916 | 397.0984497 |
| A0A4Z2DAV8 | Elongation factor 2 | 1966.675659 | 2575.295166 | 2849.078857 | 27.07925415 | 52.44044113 | 34.78770065 |
| A0A4Z2DH19 | Tubulin alpha chain | 1176.231445 | 834.6643677 | 807.8504028 | 852.4224243 | 888.5076904 | 1070.36499 |
| A0A4Z2CU87 | Clathrin heavy chain | 165.4367065 | — | 304.9555054 | — | — | — |
| A0A4Z2DIA7 | vesicle-fusing ATPase | 111.3004074 | 144.315567 | 181.4192505 | 1462.401001 | 2166.912842 | 788.5516357 |
| A0A4Z2DQD9 | Phosphoglycerate kinase | 412.1768799 | 483.8520508 | 466.3702393 | 220.5226746 | — | 296.0124207 |
| A0A4Z2DT70 | small monomeric GTPase | 462.6289063 | 446.7044067 | 371.4232178 | 1614.640381 | 1409.147949 | 1972.010376 |
| A0A4Z2DTS6 | Calmodulin isoform 1 | 913.8101807 | 820.5231323 | 927.8510742 | — | 331.3096008 | — |
| A0A4Z2DTY1 | Pre-mRNA-processing-splicing factor 8 | 193.1989899 | 221.8353882 | 224.7041321 | — | 327.5646973 | 307.743927 |
| A0A4Z2DUG9 | Actin-1 | 36567.14844 | 41485.78906 | 44899.86719 | 263.8025208 | 292.0158691 | 261.6683044 |
| A0A4Z2DVH8 | Tyrosine-protein kinase | 112.9226456 | — | — | 52.55190659 | 242.385025 | — |
| A0A4Z2DWJ8 | GTP-binding nuclear protein | 192.5611572 | 140.6243134 | 143.4328766 | 754.7226563 | 374.4610291 | 376.5048828 |
| A0A4Z2DXW1 | 60 kDa heat shock protein | 4875.459473 | 7048.239746 | 8260.494141 | 1455.025269 | 995.9998169 | 1221.568726 |
| A0A4Z2D439 | Dynein heavy chain, cytosolic | 131.234726 | 78.84689331 | 114.1777039 | 212.8041687 | 239.5873413 | 199.9193268 |
| A1XBS3 | GTP-binding protein alpha-i subunit | 115.5202179 | — | 138.3425751 | 30598.23828 | 32029.48828 | 30421.20313 |
| C1L762 | ATP synthase subunit beta, mitochondrial | 339.5619507 | — | — | 7019.074219 | 3553.52124 | 4882.794434 |
| C1L8X4 | Actin 5C | 14479.21484 | 13567.17676 | 9984.857422 | 357.9989319 | 316.7201538 | 397.12854 |
| C1LAL7 | Histone H2B | 1776.77417 | 1811.711304 | 2115.23999 | 110.8710785 | 171.938324 | 203.4009857 |
| A0A4Z2CRX2 | Plasma membrane calcium-transporting ATPase 2 (Fragment) | 13.67759705 | 31.80566597 | 41.55389786 | — | — | — |
| C1LMD1 | Cell division control protein 42 | 1956.402588 | 1827.915039 | 1640.398315 | 1747.689941 | 1740.439819 | 1648.188232 |
| C1LN37 | Tubulin beta chain | 1375.529053 | 1818.72937 | 1821.996338 | 640.9884644 | 389.7296448 | 915.3855591 |
| C1LSK4 | Large ribosomal subunit protein eL42 | 240.7188263 | — | — | 1641.048462 | 3667.303467 | — |
| C1LUM6 | Small ribosomal subunit protein eS28 | 124.5816498 | 74.89511871 | 98.35574341 | 2134.578613 | 2035.859497 | 1934.564331 |
| C1LV98 | Small ribosomal subunit protein uS9 | 261.5776367 | 242.1666718 | 227.4645996 | 3504.943604 | 2549.126465 | 2870.491699 |
| C1LVH1 | Histone H3 | 117.5888596 | 114.9138641 | 86.314888 | 712.9081421 | 267.5316772 | 538.208374 |
| C1LX69 | Large ribosomal subunit protein uL14 | 1391.763184 | 3401.08374 | 2361.11084 | 85.64923859 | 52.68711853 | 109.4440765 |
| Q5BTM8 | Homolog of yeast nuclear protein localization 4 (Fragment) | 195.7419739 | 196.9270935 | 205.734314 | 52.03029251 | 82.60192108 | 84.33112335 |
| Q5BXN9 | SJCHGC05493 protein (Fragment) | 714.1504517 | — | 31.76224327 | 57.72928238 | 63.84432983 | 59.9144249 |
| Q5BXV3 | SJCHGC03526 protein (Fragment) | 429.342865 | 780.2762451 | 631.0042725 | 192.8305511 | 205.5913239 | 210.6844788 |

Note：PG represted protein group.
